# Adhesion and Colonization Intensity of *Staphylococcus epidermidis*, *Pseudomonas aeruginosa,* and *Candida albicans* on Smooth, Micro-Textured, and Macro-Textured Silicone Biomaterials

**DOI:** 10.3390/jfb16090322

**Published:** 2025-09-01

**Authors:** Kirils Jurševičs, Ingus Skadiņš, Jeļena Krasiļņikova, Anna Lece, Andrejs Šķesters, Eduards Jurševičs

**Affiliations:** 1 Department of Doctoral Studies, Riga Stradiņš University, LV1007 Riga, Latvia; 2 Department of Biology and Microbiology, Riga Stradiņš University, LV1007 Riga, Latvia; ingus.skadins@rsu.lv; 3 Department of Human Physiology and Biochemistry, Riga Stradiņš University, LV1007 Riga, Latvia; jelena.krasilnikova@rsu.lv; 4 Scientific Laboratory of Biochemistry, Riga Stradiņš University, LV1067 Riga, Latvia; anna.lece@rsu.lv (A.L.); andrejs.skesters@rsu.lv (A.Š.); 5 Clinic of Aesthetic Medicine of Medical Doctor Edward Yurshevich, LV1010 Riga, Latvia; lipex@lipex.lv

**Keywords:** silicone implant, biomaterial, adhesion, colonization, *Staphylococcus epidermidis*, *Pseudomonas aeruginosa*, *Candida albicans*

## Abstract

Implantable biomaterials are widely used in modern medicine, especially in orthopaedics, cardiovascular surgery, dentistry, and plastic and reconstructive surgery. The issue of the interaction of implants with body tissues and the risk of infection associated with them is one of the most studied and topical issues in medicine. It is very important to find a biomaterial that effectively combines both microbiology and tissue compatibility aspects. The aim of this research work was to determine the adhesion and colonization rates of *Staphylococcus epidermidis*, *Pseudomonas aeruginosa*, and *Candida albicans* on smooth, microtextured, and macro-textured silicone biomaterials in an in vitro study. A total of 90 silicone biomaterial samples were used, 30 for each type of biomaterial. In each of the biomaterial groups, half of the samples (*n* = 15) were used to determine the adhesion intensity and the other half to determine the colonization intensity on the active surface of the biomaterial samples. The study found that *Staphylococcus epidermidis* and *Pseudomonas aeruginosa* had the highest adhesion intensity on the macro-textured implant, while *Candida albicans* adhered best to smooth. Among the microorganisms, *Pseudomonas aeruginosa* demonstrated the highest colonization rate, followed by *Staphylococcus epidermidis* and then *Candida albicans*. The most intensive colonization of microorganisms was on the macro-textured implant, then on the micro-textured, and then on the smooth. The smooth and micro-textured implants did not show statistically significant differences in the intensity of adhesion and colonization. The biomaterials did not show pro-oxidant or anti-oxidant properties, and no lipid peroxidation was induced by the biomaterials.

## 1. Introduction

In modern medicine, artificial synthetic biomaterials—implants—are widely used in many fields. These are medical devices that are transplanted into the body and used to replace or support a damaged organ, improve functions, and find faults in the normal functioning of the body. Biomaterials are most widely used in orthopaedics, cardiovascular surgery, dentistry, and plastic and reconstructive surgery. The issue of the interaction of implants with body tissues and the associated risk of infection is one of the most relevant and most studied in medicine [1,2].

Silicone is intensively studied as an implantable material. It is a ubiquitous biomaterial, as more than 1000 medical products contain medical-grade silicone, either as a component or as a residue from the manufacturing process. For example, every syringe and needle, as well as intravenous systems, are lubricated with silicone. Silicone elastomers in their solid configuration are used as pacemaker coatings, tubing, prosthetic joints, hydrocephalus shunts, and several facial and penile implants. As with breast implants, some testicular and chin prostheses and implants are made of silicone gel and encapsulated in a silicone shell [2,3].

Silicone is a polymer composed of repeating molecules of siloxane, which consist of silicon, oxygen, and saturated alkane side chains. Polydimethylsiloxane (PDMS) is the polymer most widely used in medicine. It is a very pure polymer consisting of a silicone base with two methyl groups and is one of the most inert biomaterials used in medicine. In turn, silicone breast biomaterials with silicone gel content are hardened by exchanging the methyl side chain (CH3) for a vinyl side chain (CH-CH2), which allows the silicone chains to cross-link with each other. The outer shell of the silicone is made of fully polymerized silicone with non-crystallized silicon oxide added for strength [2,3].

Of all silicone biomaterials, silicone breast implants are the most widely used for breast augmentation and reconstruction after mastectomy. Nearly two million breast augmentation surgeries were performed worldwide in 2023, with a projected increase of five to seven percent per year. According to the International Society of Aesthetic Plastic Surgery (ISAPS), 2,174,616 women have had breast implants. From 2018 to 2022, breast implants demonstrated a constant increase. In the United States alone, 255,200 breast reconstruction surgeries after mastectomy were performed in 2022, and 85% of cases were reconstructed with silicone implants [1,4,5].

After implantation, the human body responds to the foreign body by activating the immune system, which forms a capsule of collagen fibres around the implant. Capsule formation is a normal tissue response, but it can cause problems when it contracts around the implant, deforming the breast implant and causing soreness. This condition is defined as capsular contracture. The mechanisms behind capsular contracture are not clearly understood, but it is thought that the arrangement of collagen fibres plays a key role in the formation of capsular contracture, and by disrupting this arrangement, the incidence and severity of capsular contracture will decrease [6,7,8].

In the 1970s, when implant texturing became popular, it was concluded that it could affect capsule formation by disrupting the arrangement of fibres around the implant and the adhesion of tissue to the biomaterial. Tissue ingrowth into the textured biomaterial disrupts the fibre arrangement, which has been associated with reduced clinically relevant capsule formation. By contrast, a smooth silicone implant forms an unadhered dense capsule with well-organised and ordered collagen fibres [1,8,9,10].

**Figure 1:**
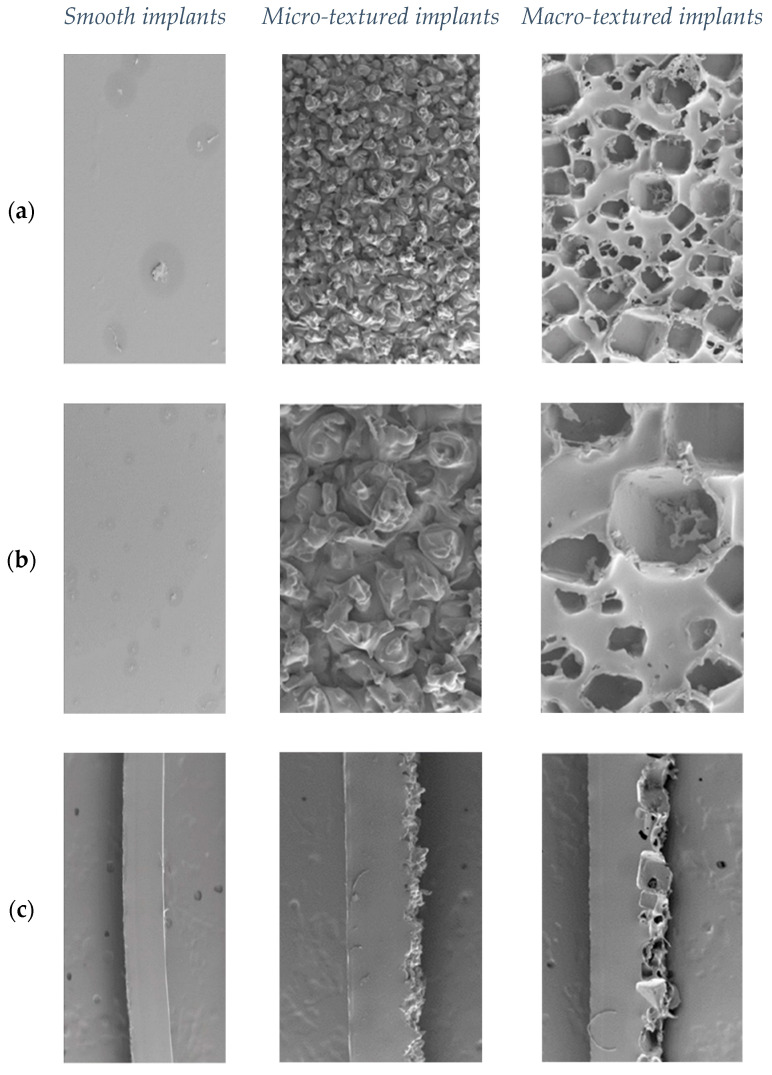
Scanning electron microscope images of smooth, micro-textured, and macro-textured silicone biomaterials. From upper to lower—(**a**) top view 40× magnification, (**b**) top view 100× magnification, (**c**) cross-section [11].

Different types of implant surfaces are created using different technologies. Silicone implant textures are classified by the surface area per 5 mm radius disk. A perfectly smooth disk with a radius of 5 mm has a surface area of 79 mm^2^. Smooth or nanotextured implants have a surface area of 80 to 100 mm^2^, micro-textured—from 100 to 200 mm^2^, macro-textured—from 200 to 300 mm^2^, and macro-textured-plus—more than 300 mm^2^. A specific texture is difficult to detect for a smooth implant; it is made by dipping the form in liquid silicone several times to create multiple layers and allowing it to cure in a laminar flow oven (Figure 1). For micro-textured implants, the outer capsule is covered with pores 70 to 150 µm in diameter and 40 to 100 µm in height. This can be obtained by “imprint stamping” of uncured silicone shell into the polyurethane foam (Figure 1). For macro-textured implants, these parameters are 600 to 800 in diameter and 150 to 200 in height. This can be obtained using the “loss-salt” technique, when the uncured silicone outer shell is being pressed by a layer of salt crystals (Figure 1). Compared to a completely smooth surface, the smooth implant area increased by 8%, for micro-textured by 60% and for macro-textured by 171% [11,12].

Despite the advantages offered by the use of implants, they are characterised by some problems, such as impaired integration, inflammatory processes, host rejection syndrome, and bacterial infection, which is the main cause of loss of the implant. Bacteria and fungi can attach and form biofilms on foreign bodies such as central and peripheral catheters, urinary catheters, breast and orthopaedic implants, which will lead to the development of infection. Biofilm-forming microorganisms are more resistant to antimicrobial therapy due to horizontal gene transfer, which further aggravates the course of infection. Biofilms require a surface, moisture, and nutrients to grow. The most common biofilm causative agents on implant surfaces are Gram-positive (especially *Staphylococcus aureus*), Gram-negative (*Enterococcus* spp., *Pseudomonas aeruginosa*), and *Candida* spp. Such biofilms have a tendency to cause systemic infections and are often associated with repeated manipulation or surgery and removal of the implanted device [3,13,14,15].

Silicone biomaterial contamination is a common complication after breast augmentation surgery, occurring in between 0.73 and 2.5% of cases. The incidence of implant infection is highly clinic-dependent and in some institutions can reach 53% of cases. Implant infections are more common in breast reconstructive surgery compared to breast augmentation. Bacterial implant contamination can cause both acute postoperative consequences and long-term complications, which can lead to prolonged and severe antibacterial therapy, breast implant failure, repeat surgeries, and a serious decrease in the patient’s quality of life [16,17,18].

The most common causes of implant-related infections, according to the literature, are Gram-positive microorganisms, such as coagulase-negative staphylococci, *Staphylococcus aureus*, and streptococci. A study in France noted that Gram-negative bacilli, especially *Pseudomonas aeruginosa*, were second only to *S. aureus* as the causative agents of implant infections. *Mycobacterium fortuitum* is most often associated with breast implant infections, as well as some other nontuberculous mycobacteria including *Mycobacterium avium, Mycobacterium abscessus* and *Mycobacterium chlonae*. In turn, fungal infections can also cause systemic manifestations and often indicate central venous catheters as the gateway of entry, where *Candida* spp. has been described as the second most common cause of infections with an incidence of 25% in patients with central venous catheters and parenteral nutrition [16,17,18,19,20].

The pathogenesis of silicone biomaterial-related infections starts with bacterial adhesion to other cells and implant surfaces using different cell wall proteins, followed by conversion of the microorganism into an attached form and secretion of an extracellular polymeric substance that forms a barrier between bacterial microcolonies and the extracellular environment. In animal models, the subcutaneous presence of foreign material reduces the minimum number of *S. aureus* CFU capable of causing infection from 100,000 to 100 [17,18,20,21,22].

As previously mentioned, textured implants have a positive effect on capsule formation around the implant by reducing their density. On the other hand, literature data show that textured implants tend to have better growth of microorganisms. Textured implants demonstrated a threefold higher infection rate compared to implants with a smooth surface. In turn, infections have been associated with biofilms, leading to complications with breast implants, including capsular contracture [23,24].

Therefore, in order to reduce the risk and frequency of infection associated with implantable biomaterials, it is important to determine the ability of a specific biomaterial to attract and colonize common infectious agents, both bacterial and fungal, on its surface.

The aim of the work was to investigate the adhesion and colonization intensity of *Staphylococcus epidermidis*, *Pseudomonas aeruginosa*, and *Candida albicans* on smooth, micro-textured, and macro-textured silicone biomaterials in an in vitro study.

## 2. Materials and Methods

### 2.1. Biomaterials Used in the Study

Three new, sterile, previously unused silicone biomaterials with different surface structures were used for this study. Each implant had a silicone outer capsule. One of the implant types had a smooth or nanotextured surface. The other type had a micro-textured surface, and the third type had a macro-textured surface. For micro-textured implants, the outer capsule is covered with pores 70 to 150 µm in diameter and 40 to 100 µm in height (Figure 1), while for macro-textured implants these parameters were 600 to 800 µm in diameter and 150 to 200 µm in height, respectively (Figure 1). Smooth implants (Figure 1) have an area increased by 8% compared to a completely smooth surface, micro-textured implants by 60%, and macro-textured implants by 171%. The internal contents of the implants (silicone in the case of these implants) were evacuated under sterile conditions, the internal surface was cleaned of its remains, and then the external surface was cut into squares with a side of 1 cm, thus the surface area for each sample was 1 cm^2^. A total of 40 cm^2^ was cut from each implant. Each group of samples was packaged individually and sterilized again in an autoclave using a standard programme [11,12].

### 2.2. Culture of Microorganisms Used in the Study

Three different microorganisms were selected for the experiment. Gram-positive *Staphylococcus epidermidis* ATCC 12228 and Gram-negative *Pseudomonas aeruginosa* ATCC 27853, but *Candida albicans* ATCC 10231 were presented from the fungi kingdom.

The microorganisms were taken from pure cultures under sterile conditions. Suspensions of 2 mL were prepared with each of the microorganisms in physiological saline (0.9% NaCl); each suspension contained 10^2^ CFU (colony-forming units). The samples of each implant type were individually placed into separate tubes with suspension and divided into the groups.

### 2.3. Determination of Adhesion and Colonization Intensity of Studied Microorganisms on the Smooth, Micro-Textured, and Macro-Textured Silicone Biomaterial

**Scheme 1:**
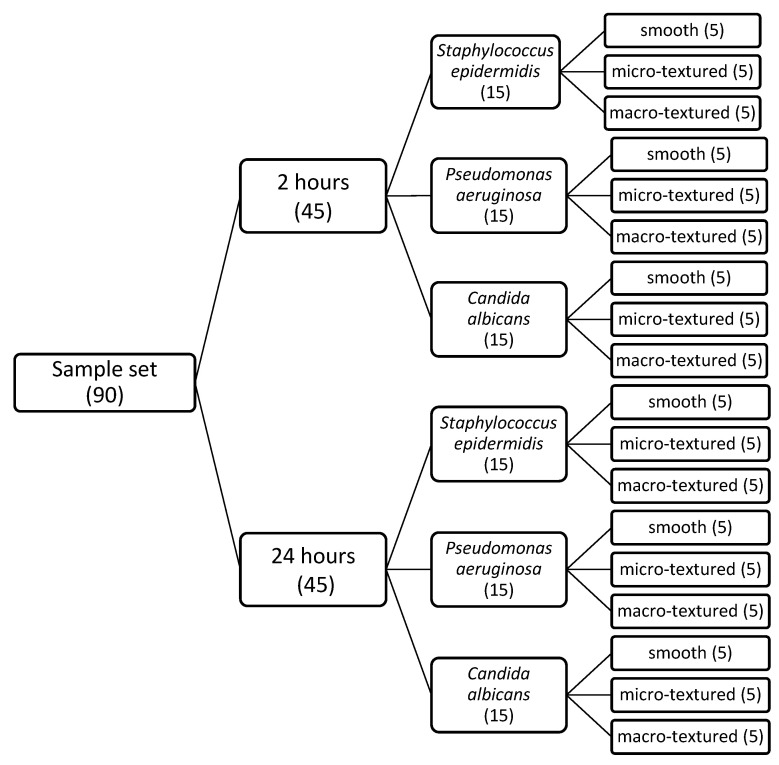
Distribution of testing groups. The number of samples in each group is given in brackets.

Ninety tubes were prepared with suspensions of *Staphylococcus epidermidis*, *Pseudomonas aeruginosa*, and *Candida albicans* at a concentration of 10^2^ CFU/mL, 30 for each micro-organism. A total of 30 samples from each implant were used. Each individual piece of implant was inoculated into the respective culture medium according to the scheme. Two independent groups were created, with different incubation times of 2 and 24 h (Scheme 1). The two-hour incubation group was designed to check the adhesion of the microorganisms to the respective implant, and the 24-h group to observe the colonization intensity. The tubes containing the samples were incubated at 37 °C.

Since implants in vivo have only one active surface—the outer capsule, but in this in vitro experiment, both the outer and inner surfaces of the implants were in contact with microorganisms, the inner surface had to be cleared of them. Following the principles of antiseptics and disinfection, each implant sample was removed from the suspension and, after visualizing its inner surface, it was wiped with a disposable ethyl alcohol-containing napkin, while the outer surface was left intact. The implant samples were then rinsed with saline to wash away unattached microorganisms. Treated implant samples were placed in new tubes with 2 mL of 0.9% NaCl saline, and the old tubes were discarded.

The hermetically sealed tubes with implants were placed in a Cole-Palmer Ultrasonic Cleaner bath filled with water. They were irradiated at a frequency of 44 kHz for one minute in order to remove attached microorganisms from the implant surface and mix them in physiological fluid. Next, the microorganisms free from the implant were homogeneously mixed into the saline for one minute using the Biosan Vortex V-1 plus apparatus (Riga, Latvia). Inoculation was performed from each tube, observing the principles of antiseptics and disinfection. A micropipette was used to take 200 µL of the newly formed suspension and introduce it into separate Petri plates.

The experiment required the use of different incubation media. Trypticase soy agar was prepared for bacteria, and Sabouraud agar for fungi. Further identifying each medium, they were incubated for 72 h in different thermal chambers with different temperature regimes—37 °C for bacteria and 22 °C for fungi.

In each medium, the number of CFU per square centimetre was counted at five different locations. The average CFU density per 1 cm^2^ was calculated using the following formula:

$$CFU=\pi r^{2}\;*\;X\;*\;K$$

where

CFU—absolute number of colonies forming units per 1 cm^2^ of implant;

πr^2^—area of the Petri plate with a radius of 4.25 cm—total area of 56.7 cm^2^;

*X*—arithmetic mean of the number of colonies per Petri plate;

*K*—dilution factor 10, as 200 µL of 2 mL was taken from each tube.

The formula was used to obtain a figure representing the absolute number of CFU of microorganisms attached to the respective implant sample.

### 2.4. Additional Testing on Antioxidant Properties and Lipid Peroxidation

A total of 3 samples from each implant type, prepared as described above (Section 2.1), were used. Each individual piece of implant was placed into a sterile tube containing 2 mL of sterile 0.9% NaCl saline (a total of 9 tubes were prepared) and stored at 37 °C for 72 h. After 24 h and at 72 h, samples for (1) antioxidant activity using the ABTS cation-radical test and (2) lipid peroxidation using the thiobarbituric acid reactive substances (TBARS) assay were taken. Antioxidant status was assessed using the Total Antioxidant Status commercial assay kits (Randox Laboratories Ltd., Crumlin, UK) adapted to the RX Imola™ automated chemistry analyser (Randox Laboratories Ltd., Crumlin, UK) following the manufacturer’s instructions. TBARS was detected using OxiSelect™ TBARS (MDA Quantitation) Assay kit, Cell Biolabs, Inc., San Diego, CA, USA, according to the manufacturer’s instructions. The absorption spectrum was measured using a multimodal microplate reader, SPARK, TECAN, Grödig, Austria.

### 2.5. Statistical Analysis

The results obtained were compiled in Microsoft Office Excel, transformed into IBM SPSS Statistics 22, and further statistically processed. Statistical analysis of groups was performed using the Mann-Whitney U test and the Chi-Square test, which are suitable for analysing non-parametric data, as the number of CFU in each group does not follow a normal distribution. *p*-value < 0.05 was set as a statistically significant level. Data are presented as mean ± standard deviation and graphically.

## 3. Results

Numbers of colony-forming units for adhesion and colonization of studied microorganisms on different surfaces of silicone biomaterial are provided in the Supplement Tables below (Tables S1 and S2).

No *S. epidermidis* adherence was observed on one of the five plates of the implant biomaterials with a smooth surface and on two of the five plates with a micro-textured surface. No *P. aeruginosa* adherence was observed on one of the five plates of the implant biomaterials for each of the different tested surfaces. No *C. albicans* adherence was observed on three of the five plates of the implant biomaterials with smooth surfaces and on four of the five plates for micro- and macro-textured surfaces.

Neither any antioxidant activity nor lipid peroxidation signs were detected in the prepared samples of smooth, micro-textured, and macro-textured implants stored at 37 °C for 24 or 72 h.

## 4. Discussion

In order to assess the differences in adhesion and colonization rates between the different silicone biomaterials in each microorganism group, the null hypothesis that there is no statistically significant difference in adhesion and colonization between the biomaterials was established.

**Figure 2:**
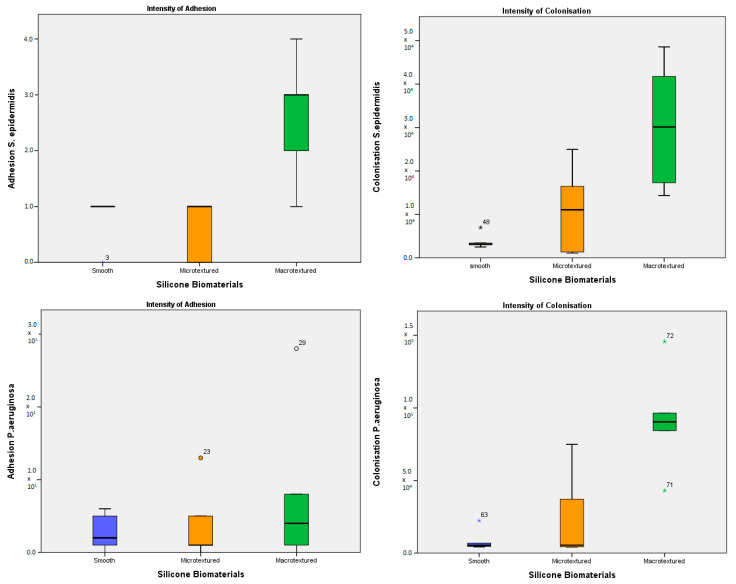

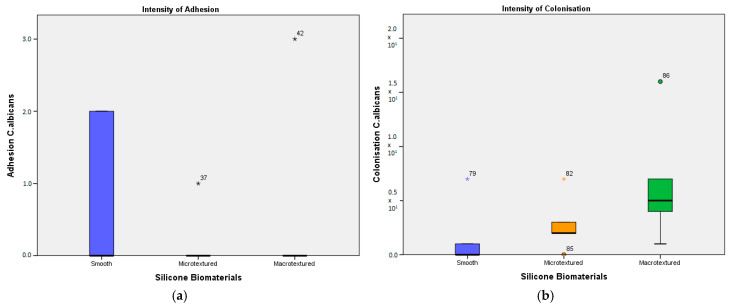
Adhesion (**a**) and colonization (**b**) intensity of *Staphylococcus epidermidis*, *Pseudomonas aeruginosa*, and *Candida albicans* on smooth (blue), micro-textured (orange), and macro-textured (green) silicone implant biomaterials.

The analysis performed for adhesion intensity of *S. epidermidis* showed that there was no statistically significant difference between smooth and micro-textured implants (*p* = 1.000) and between smooth and macro-textured implants (*p* = 0.069), but the difference was revealed between micro-textured and macro-textured implants (*p* = 0.021) (Figure 2a). For the colonization rate of *S. epidermidis*, no difference between smooth and microtextured implants (*p* = 1.000) was detected, while there was a statistically significant difference between smooth and macro-textured implants (*p* = 0.027) and a trend towards the difference between micro-textured and macro-textured implants (*p* = 0.121) (Figure 2b). Thus, the macro-textured implants were more suitable for both adhesion and colonization for *S. epidermidis*. Similar results were published by Lee and colleagues. They showed that the number of tested *S. aureus* and *S. epidermidis* attached to the smooth and nanotextured surface implants was significantly lower than that on the macro-textured surface when the incubation time was 24 h [25]. This was aligned with results obtained by Jacombs and colleagues, which demonstrated that textured implants develop a significantly higher load of bacterial biofilm in comparison with smooth implants was studied in a human strain of *S. epidermidis* [26]. Further findings confirm that adhesion, growth, colonization, and biofilm formation of Staphylococci are more expressed on the implant surface with increased texture [27].

Assessing the intensity of *P. aeruginosa* adhesion, it was found that there is no statistically significant difference between silicone biomaterials. In absolute terms, it was observed that the difference between implant types was not pronounced, but relatively more CFU adhered to the macro-textured implant surface (Figure 2a). Not expressed differences in adhesion still allowed the microorganism to develop the highest colonization rate on macro-textured implant type, less pronounced on micro-textured (significant, *p* = 0.022) biomaterial (Figure 2b). The published results obtained during similar in vitro experiments confirm our findings: no statistically significant differences were revealed in the numbers of *P. aeruginosa* CFU recovered from the nanotextured, smooth, and macro-textured implants, while these numbers were lower for the nanotextured and smooth implants [25]. Other investigators revealed an absence of a significant difference between smooth and textured surfaces and showed variable interactions tested *P. aeruginosa* that needed further molecular analysis to assess the nature of adherence [28].

Evaluating the adhesion of *C. albicans*, contradictory results were observed. It was found that there is no statistically significant difference between the biomaterials, in spite of the fact that, in absolute numbers, the most *C. albicans* adhered directly to the smooth implant. However, the adhesion of *C. albicans* was detected only on 2 of 5 plates for smooth implant and only on 1 of 5 plates for both micro- and macro-textured, which definitely impacts the result. Probably, this can be regarded as a mistake or an experimental disturbance. Similar to the adherence rate, the colonization was detected only in 2 of 5 plates for the smooth implant. In absolute numbers, a greater difference was observed between the tested implants. With poor adherence intensity, *C. albicans* mostly colonised on the macro-textured implant, followed by the micro-textured and then the smooth one (Figure 2b).

Comparing the differences in the adhesion and colonization intensity of *S. epidermidis, P. aeruginosa,* and *C. albicans* on smooth, micro-textured, and macro-textured silicone biomaterials using the Mann-Whitney U test shows a statistically significant difference in all groups, except for *C. albicans* on smooth and micro-textured biomaterials.

Taken together, the surface of the macro-textured implant turned out to be particularly favourable for adhesion and colonization of the tested microorganisms, which was expected. Therefore, in order to minimise the possible biofilm formation on the surface of silicone implants, the smooth and micro-textured biomaterial looks preferable for use in breast surgery. Though according to the previously reviewed data, the highest percentage of capsular contracture cases, the most common complications in surgical interventions, in primary augmentation patients, was for smooth implants [29], which contradicts the conclusion made. The process of capsular contracture development depends on various factors, including physiological interaction between implant material and host organism, as well as bacterial film formation, which, in turn, is affected by the species of microorganism and the surface of the implant, and it is difficult to select one of them. Numerous clinical studies illustrated a significant correlation between the presence of bacterial colonization/biofilm and capsular contracture of breast implants [30]. From this point of view, micro-textured biomaterial is more suitable for use in breast surgery. On the other hand, the situation concerning fungi is not so ambiguous, and further studies with a larger number of samples are necessary to reduce the impact of data dispersion on the final results.

During a previous study of mammary gland surgery, the alteration of the antioxidant status of blood in patients undergoing breast augmentation was noted [31]. It was interesting to reveal whether the implant material which has a different surface and whether the manufacturing technology itself has antioxidant activity or prooxidant properties. Neither any antioxidant activity nor lipid peroxidation signs were proved in this in vitro experiment, indicating the inertness of the material studied.

Like any other research, this study has its own limitations. The main problem was moderate data dispersion in each subgroup, which could be solved with a larger quantity of tested units in each subgroup. Another issue that can be mentioned is counting the CFU on each Petri plate. We used a visual method with magnifying loupes over graph paper; other, more precise methods could be used in future tests. More implants in the same group can be tested in the future to see the consistency of the results. The cleaning of the inner side of the implant can be improved to reduce the risk of contamination of the “clean” side and decontamination of the field of experiment.

## 5. Conclusions

Summarizing the obtained results, it can be concluded that the highest adhesion intensity of *Staphylococcus epidermidis* and *Pseudomonas aeruginosa* was on the macro-textured implant, while *Candida albicans* adhered best to the smooth.

Among microorganisms, the highest bacterial growth was demonstrated by *Pseudomonas aeruginosa*, followed by *Staphylococcus epidermidis* and then *Candida albicans*. The most intensive colonization of microorganisms was found on the macro-textured implant, then on the micro-textured, and on the smooth. Smooth and micro-textured implants did not show statistically significant differences in the adhesion and colonization intensity, which allows us to assume that in surgeries, where the formation of a dense capsule around the silicone biomaterial is not favourable, micro-textured implants are the best option, as they combine both microbiological and tissue compatibility aspects. The biomaterials did not show antioxidant properties, and lipid peroxidation induced by the biomaterials was not present.

## Data Availability

The original contributions presented in the study are included in the article/Supplementary Materials, further inquiries can be directed to the corresponding author.

## Supplementary Materials

The following supporting information can be downloaded at: https://www.mdpi.com/article/10.3390/jfb16090322/s1, Table S1: Intensity of Staphylococcus epidermidis, Pseudomonas aeruginosa and Candida albicans adhesion on smooth, micro-textured and macro-textured silicone implant biomaterial.; Table S2: Intensity of *Staphylococcus epidermidis*, *Pseudomonas aeruginosa* and *Candida albicans colonization* on smooth, micro-textured and macro-textured silicone implant biomaterial.

## Abbreviations

The following abbreviations are used in this manuscript:

ATCC	American Type Culture Collection
CFU	Colony Forming Units
ISAPS	International Society of Aesthetic Plastic Surgery
MDA	Malondialdehyde
TBARS	Thiobarbituric Acid Reactive Substances

## References

[1] Ratner B.D., Bryant S.J. (2004). Biomaterials: Where We Have Been and Where We Are Going. Annu. Rev. Biomed. Eng..

[2] King T.W., Butler C.E. (2017). Implants and Biomaterials. Plastic Surgery.

[3] Pecquet Goad M.E., Goad D.L. (2013). Biomedical Materials and Devices. Hachek and Rousseaux’s Handbook of Toxicologic Pathology.

[4] The Toronto Plastic Surgeons Breast Augmentation (Implants) Statistics 2023. https://torontoplasticsurgeon.com/blog/breast-augmentation-implants-statistics-2023.

[5] ISAPS Global Survey 2023. Full Report and Press Release in English..

[6] Bui J.M., Perry T., Ren C.D., Nofrey B., Teitelbaum S., Van Epps D.E. (2015). Histological Characterization of Human Breast Implant Capsules. Aesthetic Plast. Surg..

[7] Efanov J., Giot J., Fernandez J., Danino M. (2017). Breast-implant texturing associated with delamination of capsular layers: A histological analysis of the double capsule phenomenon. Ann. Chir. Plast. Esthet..

[8] Hakelius L., Ohlsen L. (1992). A clinical comparison of the tendency to capsular contracture between smooth and textured gel-filled silicone mammary implants. Plast. Reconstr. Surg..

[9] Danino M.A., Efanov J.I., Dimitropoulos G., Moreau M., Maalouf C., Nelea M., Izadpanah A., Giot J.-P. (2018). Capsular Biofilm Formation at the Interface of Textured Expanders and Human Acellular Dermal Matrix: A Comparative Scanning Electron Microscopy Study. Plast. Reconstr. Surg..

[10] Derby B.M., Codner M.A. (2015). Textured Silicone Breast Implant Use in Primary Augmentation. Plast. Reconstr. Surg..

[11] Atlan M., Nuti G., Wang H., Decker S., Perry T. (2018). Breast implant surface texture impacts host tissue response. J. Mech. Behav. Biomed. Mater..

[12] Webb L.H., Aime V.L., Do A., Mossman K., Mahabir R.C. (2017). Textured Breast Implants: A Closer Look at the Surface Debris Under the Microscope. Plast. Surg..

[13] Kumeria T., Mon H., Aw M.S., Gulati K., Santos A., Griesser H.J., Losic D. (2015). Advanced biopolymer-coated drug-releasing titania nanotubes (TNTs) implants with simultaneously enhanced osteoblast adhesion and antibacterial properties. Colloids Surf. B Biointerfaces.

[14] Song Z., Borgwardt L., Høiby N., Wu H., Sørensen T.S., Borgwardt A. (2013). Prosthesis infections after orthopedic joint replacement: The possible role of bacterial biofilms. Orthop. Rev..

[15] Yoda I., Koseki H., Tomita M., Shida T., Horiuchi H., Sakoda H., Osaki M. (2014). Effect of surface roughness of biomaterials on *Staphylococcus epidermidis* adhesion. BMC Microbiol..

[16] Lalani T. (2018). Breast Implant Infections: An Update. Infect. Dis. Clin. N. Am..

[17] Seng P., Bayle S., Alliez A., Romain F., Casanova D., Stein A. (2015). The microbial epidemiology of breast implant infections in a regional referral centre for plastic and reconstructive surgery in the south of France. Int. J. Infect. Dis..

[18] Washer L.L., Gutowski K. (2012). Breast Implant Infections. Infect. Dis. Clin. N. Am..

[19] Macadam S.A., Mehling B.M., Fanning A., Dufton J.A., Kowalewska-Grochowska K.T., Lennox P., Anzarut A., Rodrigues M. (2007). Nontuberculous Mycobacterial Breast Implant Infections. Plast. Reconstr. Surg..

[20] Oliveira W., Silva P., Silva R., Silva G., Machado G., Coelho L., Correia M. (2018). *Staphylococcus aureus* and *Staphylococcus epidermidis* infections on implants. J. Hosp. Infect..

[21] Zimmerli W., Waldvogel F.A., Vaudaux P., Nydegger U.E. (1982). Pathogenesis of Foreign Body Infection: Description and Characteristics of an Animal Model. J. Infect. Dis..

[22] Zimmerli W., Lew P.D., Waldvogel F.A. (1984). Pathogenesis of foreign body infection. Evidence for a local granulocyte defect. J. Clin. Investig..

[23] Khavanin N., Clemens M.W., Pusic A.L.M., Fine N.A., Hamill J.B., Kim H.M.S., Qi J.M., Wilkins E.G.M., Kim J.Y.S.M. (2017). Shaped versus Round Implants in Breast Reconstruction: A Multi-Institutional Comparison of Surgical and Patient-Reported Outcomes. Plast. Reconstr. Surg..

[24] Munhoz A.M., Clemens M.W.M., Nahabedian M.Y.M. (2019). Breast Implant Surfaces and Their Impact on Current Practices: Where We Are Now and Where Are We Going?. Plast. Reconstr. Surg. Glob. Open.

[25] Lee J.H., Ryu J.Y., Lee J.S., Choi K.Y., Chung H.Y., Cho B.C., Kim K., Lee Y.J., Jin H.K., Bae J.-S. (2022). Effect of Breast Silicone Implant Topography on Bacterial Attachment and Growth: An *In Vitro* Study. In Vivo.

[26] Jacombs A., Tahir S., Hu H., Deva A.K., Almatroudi A., Wessels W.L.F., Bradshaw D.A., Vickery K. (2014). In Vitro and In Vivo Investigation of the Influence of Implant Surface on the Formation of Bacterial Biofilm in Mammary Implants. Plast. Reconstr. Surg..

[27] Schoberleitner I., Baier L., Lackner M., Zenz L.-M., Coraça-Huber D.C., Ullmer W., Damerum A., Faserl K., Sigl S., Steinkellner T. (2024). Surface Topography, Microbial Adhesion, and Immune Responses in Silicone Mammary Implant-Associated Capsular Fibrosis. Int. J. Mol. Sci..

[28] Nam S.Y., Zhang X., Faruq O., Chien P.N., Dönmez N., Heo C.Y. (2021). An Impact of Different Silicone Breast Implants on the Bacterial Attachment and Growth. J. Biomater. Nanobiotechnol..

[29] Shauly O., Gould D.J., Patel K.M. (2018). Microtexture and the Cell/Biomaterial Interface: A Systematic Review and Meta-Analysis of Capsular Contracture and Prosthetic Breast Implants. Aesthetic Surg. J..

[30] Ajdic D., Zoghbi Y., Gerth D., Panthaki Z.J., Thaller S. (2016). The Relationship of Bacterial Biofilms and Capsular Contracture in Breast Implants. Aesthetic Surg. J..

[31] Jurševičs K., Jurševičs E., Krasiļņikova J., Šķesters A., Lece A., Skadiņš I. (2024). Antioxidant Status in Patients after Breast Mastopexy and Augmentation. Medicina.

