# Supplementary material for: Adhesion and Colonization Intensity of Staphylococcus epidermidis, Pseudomonas aeruginosa, and Candida albicans on Smooth, Micro-Textured, and Macro-Textured Silicone Biomaterials
Source: J Funct Biomater. 2025 Sep 1;16(9):322. doi: 10.3390/jfb16090322 (PMC12471252; doi:10.3390/jfb16090322)
Supplement: Supplementary file 1 [file jfb-16-00322-s001.zip › jfb-3752635-supplementary.pdf]

Table S1. Intensity of *Staphylococcus epidermidis, Pseudomonas aeruginosa* and *Candida albicans* adhesion on smooth, micro-textured and macro-textured silicone implant biomaterial.

**Type of silicone biomaterial**	**Number of colonies[1], CFU/cm2**	**Number of colonies, extreme values[2], CFU/cm2**
Staphylococcus epidermidis		
smooth	453.96 ± 253.77	0 – 567
micro-textured	340.47 ± 310.8	0 - 567
macro-textured	794.43 ± 310.8	567 - 1135
Pseudomonas aeruginosa		
smooth	453.96 ± 253.77	0 - 567
micro-textured	1134.9 ± 1134.9	0 - 2837
macro-textured	2383.29 ± 3092.47	0 - 7377
Candida albicans		
smooth	340.47 ± 507.54	0 - 1135
micro-textured	113.49 ± 253.77	0 - 567
macro-textured	226.98 ± 507.54	0 - 1135

[1] Data are presented as mean value ± SD.

[2] Data are presented as min value – max value.

Table S2. Intensity of *Staphylococcus epidermidis, Pseudomonas aeruginosa* and *Candida albicans* colonization on smooth, micro-textured and macro-textured silicone implant biomaterial.

Type of silicone biomaterial	Number of colonies[1], CFU/cm2	Number of colonies, extreme values[2], CFU/cm2
Staphylococcus epidermidis		
smooth	457 591.82 ± 216 159.64	299 614 – 837 556
micro-textured	1 318 300.24 ± 1 224 070.28	129 379 - 2.996 137
macro-textured	3 651 201.39 ± 1 788 277.85	1.722 779 - 5.828 848
Pseudomonas aeruginosa		
smooth	1.039.114,76 ± 926.411,58	510 70 - 2.682 904
micro-textured	3.019.288,88 ± 3.738.924,8	490 277 - 8.988 411
macro-textured	11.039.402,65 ± 4.392.825,44	5.168 336 - 17.486 545
Candida albicans		
smooth	10895,04 ± 20765,88	47 666 - 510 705
micro-textured	16342,56 ± 18397,99	0 – 47 666
macro-textured	36770,77 ± 43760,67	6 809 – 108 950

[1] Data are presented as mean value ± SD.

[2] Data are presented as min value – max value.
